# Risk of recurrent ischemic stroke after non-cardioembolic ischemic stroke in England and Denmark

**DOI:** 10.1007/s00415-026-13872-4

**Published:** 2026-05-23

**Authors:** David Gaist, Antonio Gonzáléz-Pérez, Kristian Tore Jørgensen, Birgit Bjerre Høyer, Sören Möller, Kristina Karlsdotter, Luke Bamber, Jason Xeni, Deborah Lowe, Mukul Sharma, Luis Alberto Garcia Rodriguez

**Affiliations:** 1 Research Unit for Neurology, Odense University Hospital, University of Southern Denmark, J.B.Winsløws Vej 4, 5000 Odense C Odense, Denmark; 2 Centro Español Investigación Farmacoepidemiológica, Madrid, Spain; 3 Faculty of Health Sciences, Universidad Loyola Andalucía, Seville, Spain; 4 Bayer A/S, Copenhagen, Denmark; 5 Open Patient Data Explorative Network (OPEN), Odense University Hospital, Odense, Denmark; 6 Department of Public Health, University of Southern Denmark, Odense, Denmark; 7 Bayer Hispania, S.L.U., Barcelona, Spain; 8 Bayer AG, Wuppertal, Germany; 9 Bayer Inc, Mississauga, ON Canada; 10 Wirral University Teaching Hospital NHS Foundation Trust, Wirral, UK; 11 Department of Medicine, McMaster University and Population Health Research Institute, Hamilton, ON Canada

**Keywords:** Recurrent ischemic stroke, Non-cardioembolic ischemic stroke, Real-world evidence, Severity, Unmet needs

## Abstract

**Background:**

Recurrent ischemic stroke (IS) can occur following a non-cardioembolic IS (NCIS), despite following secondary prevention guidelines. We quantified recurrent IS risk following discharge from first-ever NCIS in clinical practice.

**Methods:**

Adult patients with first-ever NCIS were identified in England (January 2012–February 2020) and Denmark (January 2012–December 2021) and followed through March 2021 (England) and August 2022 (Denmark). Primary outcome was recurrent IS (incidence rates [IRs] per 100 person years at 12 months and over total follow-up). Cumulative hazard per 100 person years with 95% confidence intervals (CIs) was estimated. Stroke severity at index and recurrent IS were compared in Danish patients.

**Results:**

Overall, 52,419 English and 62,501 Danish patients were included (respective mean follow-ups: 3.0 and 3.9 years). Recurrent IS events totaled 5857 in England, 9489 in Denmark. IRs/100 person years were similar (England: 3.74 [95% CI 3.64–3.84]; Denmark: 3.87 [95% CI 3.79–3.95]), and highest in the first year (England: 7.39 [95% CI 7.14–7.65]; Denmark: 7.96 [95% CI 7.73–8.20]). Five-year cumulative hazard of recurrent IS was 16.53 (95% CI 16.06–17.01) in England, 18.05 (95% CI 17.64–18.47) in Denmark. Among Danish cases with severity data (n = 5540), versus index strokes, recurrent IS events were more often moderate (13.84% vs 19.01%) or severe (3.57% vs 7.92%).

**Conclusions:**

Recurrent IS after NCIS was highest in the first year and remained substantial over 5 years. In Denmark, recurrent IS severity was worse than index events. These findings underscore unmet needs for improved secondary stroke prevention.

**Supplementary Information:**

The online version contains supplementary material available at 10.1007/s00415-026-13872-4.

## Introduction

Stroke is a leading cause of mortality and morbidity worldwide [1, 2], and ischemic stroke (IS) is the most prevalent stroke subtype [3]. Survivors often experience physical, psychiatric, and psychological impairments and fear of stroke recurrence has been reported as an anxiety-provoking stimulus in stroke survivors, which may further complicate recovery [4–8]. Combined with these long-lasting and complex challenges, the continual rise in global stroke burden over the past 30 years, partly attributed to an aging population [9], highlights the need for broader understanding of stroke recurrence, especially among high-risk groups, to inform development of effective prevention strategies.

Despite guideline-directed therapy such as the use of aspirin and clopidogrel [10], the risk of a recurrent IS remains substantial in both the first year of the initial event and the long term (within 5 years) [11]. Recurrent IS events can lead to worse outcomes and higher healthcare costs than index strokes [12–14]. However, many studies examining recurrent IS are outdated or do not differentiate IS subtypes nor etiology [15–17]. This limits the ability to identify personalized strategies for populations with distinct pathophysiology, such as those with non-cardioembolic IS (NCIS). Other studies reporting recurrent IS include data that predate advances in secondary stroke prevention [10, 18], such as the widespread adoption of short-term dual antiplatelet therapy for NCIS [10, 19, 20]. There is a lack of contemporary observational studies of recurrent IS stratified by cause, particularly in patients with NCIS receiving guideline-recommended therapy.

Most IS events are non-cardioembolic and have unique etiology, necessitating specific management approaches when compared with cardioembolic strokes [10, 21]. Understanding recurrence risk and contributing factors in patients with their first NCIS is essential to inform targeted secondary prevention strategies. The Antithrombotic therapy and STroke Recurrence in patients with non-cardioembolic Ischemic Stroke (ASTRIS) study was an observational study designed to quantify the risk and severity of recurrent IS after an index NCIS in England and Denmark. Both countries have free access to healthcare, which should increase the generalizability and robustness of findings.

## Methods

This retrospective cohort study used primary care data combined with hospital data (England) and a nationwide stroke registry (Denmark) to identify two cohorts of patients with first NCIS. Patients were followed for recurrent IS as described below.

### Setting and data sources

In England, data were collated from the subset of primary care practices contributing to the Clinical Practice Research Datalink (CPRD) Aurum database [22, 23] and linked to the Hospital Episode Statistics Admitted Patient Care (HES APC) dataset and the Office for National Statistics death registration (Death Registry) [24], as described in the Supplementary Methods. In Denmark (population 5.8 million), data were retrieved from: the Danish Stroke Registry [25], the Danish National Patient Registry [26], the Danish National Prescription Registry [27], the Danish Register of Causes of Death [28], and the Danish Civil Registration System [29]. In Denmark, it is compulsory for departments evaluating patients within 7 days of stroke onset to report standardized data to the Stroke Registry [25].

### Study cohorts

The study included adult patients (aged ≥ 18 years) with a first admission for IS (International Classification of Diseases, Tenth Edition [ICD-10] code I63 as primary diagnosis) recorded in the HES APC in England between January 2012 and February 2020 and the Stroke Registry in Denmark between January 2012 and December 2021.

In the Danish cohort, patients with an atrial fibrillation (AF) diagnosis (ICD-10 I48; any position, Supplementary Table 1) any time before index IS hospitalization and up to 15 days after discharge for the index IS were excluded. In England, a similar approach was applied using both hospital and primary care data (Supplementary Table 2). Furthermore, in both cohorts, patients with oral anticoagulant use within the 90 days leading up to index stroke hospitalization were excluded (see Supplementary Methods for additional details).

### Follow-up

In both cohorts, follow-up started on Day 1 after discharge for the index IS and continued until the earliest of either the recurrent IS or the end of the study period (March 2021 for England and August 2022 for Denmark). See Supplementary Methods for additional details on censoring of follow-up.

### Outcomes

The study outcome was recurrent IS defined as the first (chronological) hospitalization recorded during follow-up as captured through the HES APC (England), Stroke Registry (Denmark), Patient Registry (Denmark), or Death Registry (England, Denmark), as shown in Supplementary Table 3. In the English cohort, we assessed frailty (classified as none, mild, moderate, or severe) based on information collected in CPRD Aurum prior to the admission for index IS and recurrent IS (see Supplementary Methods) [30, 31]. In the Danish cohort, data on stroke severity assessed on admission according to the Scandinavian Stroke Scale (SSS) score (range 0–58, a higher score indicates lower severity) were available from the Stroke Registry. These data were only available for patients whose index and recurrent events were identified through the Stroke Registry.

To establish patient characteristics at baseline and classify potential confounders and risk factors at the time of admission for the index IS, information was extracted as follows. In the English cohort, prescription data from CPRD Aurum and diagnoses recorded either in CPRD Aurum or in HES APC were used. In the Danish cohort, data from the Prescription Registry and the Patient Registry for a period from January 1995 up to and including the date of admission for the index IS in the Danish cohort were used (for codes, see Supplementary Tables 1 and 2).

### Statistical analysis

All analyses were conducted separately for each cohort. Characteristics of the study cohorts were summarized using descriptive statistics, including for information unique to each cohort, i.e. body mass index, Townsend deprivation index, number of outpatient and primary care visits, frailty index, and test results for estimated glomerular filtration rate (eGFR) in the English cohort, and marital status and severity of admission stroke in the Danish cohort.

Incidence rates (IRs) were calculated as the number of recurrent IS events during follow-up divided by the person-time at risk, with 95% confidence intervals (CIs) estimated according to Poisson distribution. IRs were estimated overall and by sex, age, duration of follow-up (< 3 months, 3–12 months, and ≥ 1 year), and in 3 time periods based on the year of the index NCIS (England: 2012–2014, 2015–2017, and 2018–2020; Denmark: 2012–2014, 2015–2017, and 2018–2021). Annual IRs for each of the 5 years of follow-up were estimated. Graphs of sex-specific Nelson–Aalen cumulative hazards during all follow-up were plotted, and cumulative hazards for successive annual periods (Year 1, Year 1 and Year 2, etc.) were estimated for up to 5 years.

To assess the association between known risk factors of IS and the risk of recurrent IS, we conducted Cox regression to estimate hazard ratios (HRs) adjusting for age (< 65, 65–75, and > 75 years) and sex. In a full model (adjusted HR [aHR]) adjustments were made for lifestyle factors, comorbidities, medications, and clinical history. Further details on adjustments made in the full model (aHR) are available in Supplementary Methods. In addition, sub-distribution HRs using Fine-Gray regression models with death as competing risk were estimated.

### Sensitivity analysis

In sensitivity analyses, and to address potential issues of misclassification of recurrent IS events that may be particularly prevalent in the early phase of follow-up (including re-admissions for causes that were incorrectly coded as recurrent IS in the primary discharge diagnosis), IRs were re-estimated after moving the start date forward to 30 days after discharge for the index IS. This analysis only included patients who had not had an outcome or been censored on Days 1–30 in the main analysis.

Among patients included in the cohort, diagnoses or procedures indicative of conditions other than AF that are known risk factors for cardioembolic IS (such as mechanical heart valves or persistent foramen ovale) were identified and classified.

## Results

After applying the exclusion criteria (Supplementary Fig. 1), the study comprised 52,419 patients in the English cohort and 62,501 in the Danish cohort. The cohorts were similar in age (English cohort: mean [standard deviation], 70.7 [14.0]; Danish cohort: 70.0 [13.2]) and sex distribution (males: England, 53.2%; Denmark, 55.9%). Other patient characteristics across both cohorts are presented in Table 1. Patients in the English cohort had a higher prevalence of most comorbidities compared with the Danish cohort, including gastrointestinal bleeds (20.6% in the English cohort vs 5.0% in the Danish cohort), chronic kidney disease (17.2% vs 2.8%), and chronic obstructive pulmonary disease (12.3% vs 7.9%). Median length of hospital stay for the index IS differed across the 2 cohorts (English cohort: median [interquartile range: IQR], 6 [2–21] days; Danish cohort: median [IQR], 3 [2–6] days). In the English cohort, based on data before the date of admission, 46.9% were fit, while 32.2%, 15.5%, and 5.3% had mild, moderate, and severe frailty, respectively (Table 1). In the Danish cohort, based on SSS score on admission, 75.5% of patients had an index IS classified as mild, 15.3% as moderate, 6.9% as severe, and 2.3% were missing.

**Table 1:** Patient characteristics across the English and Danish cohorts at the time of index admission

	England	Denmark
Characteristics	(n = 52,419)	(n = 62,501)
Sex, no. (%)		
Males	27,873 (53.2)	34,952 (55.9)
Females	24,546 (46.8)	27,549 (44.1)
Age, years, mean (SD)	70.7 (14.0)	70.0 (13.2)
Age categories, years, no. (%)		
< 65	16,082 (30.7)	20,495 (32.8)
65–75	14,458 (27.6)	18,220 (29.2)
> 75	21,879 (41.7)	23,786 (38.1)
Marital status, no. (%)		
Married or registered partnership	NR	31,425 (50.3)
Divorced	NR	10,516 (16.8)
Widow/widower	NR	12,832 (20.5)
Never married or registered partnership	NR	5389 (8.6)
Missing	NR	2339 (3.7)
Townsend deprivation index (quintiles), no. (%)		
Least deprived	10,826 (20.7)	NR
20%–40%	10,984 (21.0)	NR
40%–60%	10,662 (20.4)	NR
60%–80%	10,024 (19.1)	NR
Most deprived	9864 (18.8)	NR
BMI, kg/m^2^, no. (%)		
< 20	2792 (5.3)	NR
20–24.9	12,210 (23.3)	NR
25–29.9	15,688 (29.9)	NR
≥ 30	12,445 (23.7)	NR
Unknown	9284 (17.7)	NR
Smoking status, no. (%)		
Current	10,586 (20.2)	18,675 (29.9)
Past	20,692 (39.5)	16,831 (26.9)
Never	17,219 (32.8)	19,865 (31.8)
Missing	3922 (7.5)	7130 (11.4)
Number of hospitalizations in previous year, any diagnosis, no. (%)		
0	34,537 (65.9)	46,704 (74.7)
1	9896 (18.9)	9847 (15.8)
2	3973 (7.6)	3239 (5.2)
≥ 3	4013 (7.7)	2711 (4.3)
Number of outpatient visits in previous year, any diagnosis, no. (%)		
0	19,271 (36.8)	NR
1–2	10,591 (20.2)	NR
3–5	9451 (18.0)	NR
≥ 6	13,106 (25.0)	NR
Number of primary care visits in previous year, any diagnosis, no. (%)		
0–4	8596 (16.4)	NR
5–9	8975 (17.1)	NR
10–19	15,671 (29.9)	NR
≥ 20	19,177 (36.6)	NR
Frailty^a^		
Fit	24,582 (46.9)	NR
Mild	16,942 (32.2)	NR
Moderate	8134 (15.5)	NR
Severe	2761 (5.3)	NR
SSS score^b^ on admission for index stroke, median (IQR)	NR	52 (44–57)
SSS score, grouped^c^, no. (%)		
Mild	NR	47,182 (75.5)
Moderate	NR	9538 (15.3)
Severe	NR	4333 (6.9)
Missing	NR	1448 (2.3)
Length of hospital stay for the index stroke, days^d^		
Mean (SD)	18.1 (29.5)	6.4 (11.2)
Median (IQR)	6 (2–21)	3 (2–6)
Comorbidity at the time of the index stroke^e^, no. (%)		
Hypertension	32,119 (61.3)	41,670 (66.7)
Diabetes	12,952 (24.7)	10,208 (16.3)
Hyperlipidemia	17,058 (32.5)	17,377 (27.8)
Peripheral artery disease	3147 (6.0)	3530 (5.6)
Ischemic heart disease	11,926 (22.8)	9819 (15.7)
Congestive heart failure	2614 (5.0)	2846 (4.6)
Transient ischemic attack	4848 (9.2)	5027 (8.0)
Major bleeding event (intracranial, gastrointestinal, or genitourinary bleed)	15,314 (29.2)	9339 (14.9)
Intracranial hemorrhage (ICH, SAH, SDH, or other intracranial bleed)	1120 (2.1)	1348 (2.2)
Gastrointestinal bleed (upper, lower, or unspecified)	10,777 (20.6)	3124 (5.0)
Venous thromboembolism	3542 (6.8)	3603 (5.8)
Dementia	2879 (5.5)	2158 (3.5)
CKD^f^	9035 (17.2)	1741 (2.8)
Chronic hepatic disease	3120 (6.0)	991 (1.6)
Cancer	8942 (17.1)	9737 (15.6)
COPD	6429 (12.3)	4933 (7.9)
Disorders indicative of high alcohol use	4590 (8.8)	6342 (10.1)
Baseline eGFR (mL/min/1.73 m^2^)^g^		
≥ 90	4311 (8.2)	NR
60–89	12,926 (24.7)	NR
45–59	4924 (9.4)	NR
30–44	2669 (5.1)	NR
15–29	854 (1.6)	NR
< 15	234 (0.4)	NR
Unknown	26,501 (50.6)	NR
Use of medications at the time of the index stroke^h^		
Oral anticoagulants	443 (0.8)	593 (0.9)
Vitamin K antagonists	261 (0.5)	243 (0.4)
Direct oral anticoagulants	189 (0.4)	364 (0.6)
Antiplatelets^i^	15,129 (28.9)	15,691 (25.1)
Low-dose aspirin	11,725 (22.4)	11,648 (18.6)
Clopidogrel	4285 (8.2)	4917 (7.9)
Statins	20,227 (38.6)	15,930 (25.5)
Thiazides and other non-loop diuretics	6892 (13.1)	8774 (14.0)
Loop diuretics	4580 (8.7)	5188 (8.3)
Beta-blockers	10,554 (20.1)	10,683 (17.1)
Calcium channel blockers	13,745 (26.2)	13,240 (21.2)
ACE inhibitors and angiotensin-receptor blockers	19,225 (36.7)	21,827 (34.9)
NSAIDs	3963 (7.6)	6876 (11.0)
SSRIs	5047 (9.6)	4419 (7.1)
PPIs	16,989 (32.4)	12,104 (19.4)

*ACE* angiotensin-converting enzyme, *APC* Admitted Patient Care, *BMI* body mass index, *CKD* chronic kidney disease, *COPD* chronic obstructive pulmonary disease, *CPRD* clinical practice research datalink, *eGFR* estimated glomerular filtration rate, *HES* healthcare episode statistics, *ICH* intracerebral hemorrhage, *IQR* interquartile range, *NR* not recorded, *NSAID* non-steroidal anti-inflammatory drug, *PPI* proton pump inhibitor, *SAH* subarachnoid hemorrhage, *SD* standard deviation, *SDH* subdural hemorrhage, *SSRI* selective serotonin reuptake inhibitor, *SSS* scandinavian stroke scale

^a^Based on electronic frailty index score calculated using all available information in primary care electronic health records up to the day before admission for index IS. Based on this score, frailty was classified into fit (≤ 0.12), mild (0.13–0.24), moderate (0.25–0.36), and severe (≥ 0.36)

^b^SSS score as recorded in the Danish Stroke Registry

^c^SSS score groups are defined as follows: mild (43–58), moderate (26–42), and severe (0–25). Missing values indicate unavailable scores

^d^HES APC data in England are recorded at the finished consultant episode level, which represents the time spent under the care of a single consultant. Total duration of inpatient care was obtained by combining overlapping spells into continuous inpatient spells

^e^Any time before the date of the index stroke

^f^Based on recorded diagnoses (primary care in CPRD Aurum or hospital admissions in HES APC) compatible with Kidney Disease: Improving Global Outcomes criteria for CKD: eGFR categories G3–G5, and/or albuminuria categories A2–A3

^g^Baseline eGFR based on serum creatinine values recorded within the year before the index stroke using The Chronic Kidney Disease Epidemiology Collaboration formula

^h^Defined as a prescription supply lasting until/past the date of the index stroke or ending within the previous 90 days

^i^Also includes patients with current use of dipyridamole, ticagrelor, and prasugrel

The average follow-up was 3.0 years in the English cohort and 3.9 years in the Danish cohort. Overall, 5857 (11.2%) patients in the English cohort and 9489 (15.2%) patients in the Danish cohort had a recurrent IS of any type (including non-cardioembolic or cardioembolic). A notable portion of these events were exclusively identified through the Death Registry (England: 1140 [19.5%]; Denmark: 2386 [25.1%]; Supplementary Table 3).

**Fig. 1:**
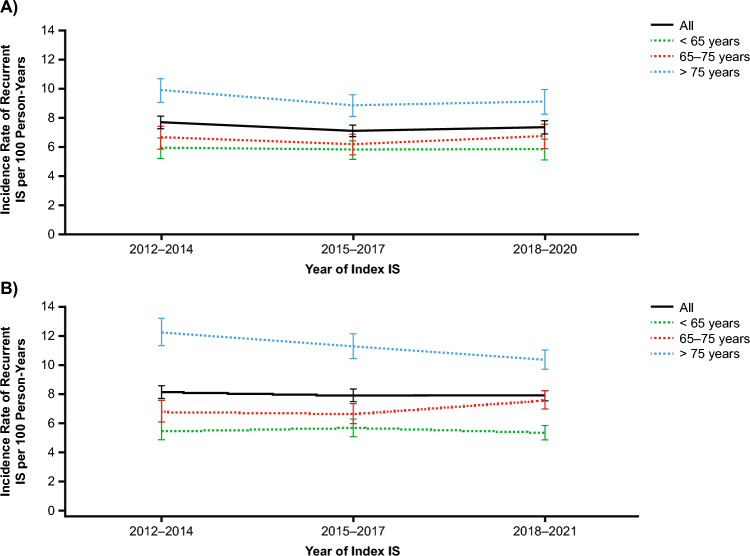
Incidence of recurrent IS by calendar period of follow-up after index non-cardioembolic IS. **A** English (n = 52,419) cohort. **B** Danish (n = 62,501) cohort. *IS* ischemic stroke

The overall IRs of recurrent IS per 100 person-years were similar between cohorts (English: 3.74 [95% CI 3.64–3.84]; Danish: 3.87 [95% CI 3.79–3.95]) (Table 2). IRs per 100 person-years were numerically slightly higher in females than males in both the English cohort (4.04 [95% CI 3.90–4.20] vs 3.49 [95% CI 3.37–3.62], respectively) and Danish cohort (3.98 [95% CI 3.86–4.10] vs 3.78 [95% CI 3.68–3.89], respectively). IRs of recurrent IS per 100 person-years were higher with age in both cohorts. IRs were highest for older patients (aged > 75 years) in both the English cohort (5.49 [95% CI 5.29–5.69)] and Danish cohort (6.26 [95% CI 6.08–6.44]), followed by those aged 65–75 years (English cohort: 3.31 [95% CI 3.15–3.48]; Danish cohort: 3.49 [95% CI 3.36–3.62]), and those aged < 65 years (English cohort: 2.47 [95% CI 2.35–2.61]; Danish cohort: 2.38 [95% CI 2.28–2.48]). Compared with analyses evaluated across all follow-up, IRs in the first year of follow-up were higher overall (English cohort: 7.39 [95% CI 7.14–7.65]; Danish cohort: 7.96 [95% CI 7.73–8.20]), and when stratified by sex and age (Table 2). During the first year of follow-up, recurrence rates were notably higher in the first month following the discharge of the index IS (Table 2A). Overall IRs in the first year of follow-up were similar across the 3 time periods from 2012 through 2021, with minimal variations over time across the 3 age groups (Fig. 1) except in the Danish cohort, where rates for the stratum of patients aged > 75 years at the time of index IS declined from 12.24 (95% CI 11.32–13.23) in 2012–2014 to 10.34 (95% CI 9.7–11.02) in 2018–2021 (*p* = 0.001, estimated using variance-weighted least squares). In contrast, recurrence rates in the English cohort for patients aged > 75 years were 9.92 (95% CI 9.11–10.78) in 2012–2014 and 9.14 (95% CI 8.31–10.03) in 2018–2021, with no significant change over time (*p* = 0.174).

**Table 2:** Incidence rate of recurrent ischemic stroke in English and Danish cohorts during the first year of follow-up and all follow-up

**A**						
	England (n = 52,419)			Denmark (n = 62,501)		
	Person-years	Events, n	IR per 100 person-years (95% CI)	Person-years	Events, n	IR per 100 person-years (95% CI)
All	43,306	3201	7.39 (7.14–7.65)	55,996	4458	7.96 (7.73–8.20)
Sex						
Females	19,856	1545	7.78 (7.40–8.18)	24,406	2010	8.24 (7.88–8.60)
Males	23,451	1656	7.06 (6.73–7.41)	31,590	2448	7.75 (7.45–8.06)
Age at index stroke, years						
< 65	14,138	832	5.88 (5.49–6.30)	19,326	1058	5.47 (5.15–5.81)
65–75	12,468	815	6.54 (6.10–7.00)	16,609	1173	7.06 (6.67–7.48)
> 75	16,701	1554	9.31 (8.85–9.78)	20,061	2227	11.10 (10.65–11.57)
Time since index IS, months^a^						
< 1	4050	1076	26.57 (25.0–28.20)	5010	1142	22.80 (21.51–24.16)
1–3	7626	752	9.86 (9.17–10.59)	9632	1262	13.10 (12.40–13.85)
≤ 3	11,676	1828	15.66 (14.95–16.39)	14,642	2404	16.42 (15.78–17.09)
3–12	31,631	1373	4.34 (4.11–4.58)	41,354	2054	4.97 (4.76–5.19)

**B**						
	England (n = 52,419)			Denmark (n = 62,501)		
	Person-years	Events, n	IR per 100 person years (95% CI)	Person-years	Events, n	IR per 100 person years (95% CI)
All	156,669	5857	3.74 (3.64–3.84)	245,343	9489	3.87 (3.79–3.95)
Sex						
Females	70,184	2838	4.04 (3.90–4.20)	106,765	4245	3.98 (3.86–4.10)
Males	86,486	3019	3.49 (3.37–3.62)	138,578	5244	3.78 (3.68–3.89)
Age at index stroke, years						
< 65	56,630	1401	2.47 (2.35–2.61)	97,246	2311	2.38 (2.28–2.48)
65–75	47,593	1577	3.31 (3.15–3.48)	75,387	2628	3.49 (3.36–3.62)
> 75	52,447	2879	5.49 (5.29–5.69)	72,710	4550	6.26 (6.08–6.44)
Time since index IS, months						
≤12	43,306	3201	7.39 (7.14–7.65)	55,996	4458	7.96 (7.73–8.20)
>12	113,363	2656	2.34 (2.25–2.43)	189,347	5031	2.66 (2.58–2.73)

*CI* confidence interval, *IR* incidence rate, IS ischemic stroke

^a^Time since index IS in days: < 1 month = < 30 days; 1–3 months = 30–90 days; < 3 months = ≤ 90 days; 3–12 months = 91–365 days

Annual IRs of recurrent IS per 100 person years were substantially lower in the second year of follow-up compared with the first year. In the English cohort, IRs were higher in Year 1 at 7.39 (95% CI 7.14–7.65) compared to 2.89 (95% CI 2.72–3.08) in Year 2. Similarly, in the Danish cohort, IRs were 7.96 (95% CI 7.73–8.20) in Year 1 and 3.41 (95% CI 3.25–3.58) in Year 2 (Table 3). In Years 3 through 5 of follow-up, annual IRs were even lower than in Year 2, although differences between years were less pronounced than the drop observed between Years 1 and 2. IRs for Years 3 through 5 can be found in Table 3. The cumulative risk of recurrent IS was higher over time in both countries. In the English cohort, the cumulative hazard per 100 person-years was 7.02 (95% CI 6.78–7.27) at Year 1 and 16.53 (95% CI 16.06–17.01) at Year 5. Similarly, in the Danish cohort, the cumulative hazard went from 6.92 (95% CI 6.71–7.14) to 18.05 (95% CI 17.64–18.47) over the same period (Table 3). Cumulative hazard of recurrent IS by sex are shown in Supplementary Fig. 2. In the English cohort, 39% of patients with recurrent IS had a higher frailty score upon re-admission compared to the time of admission for their index IS (Supplementary Table 4). In the Danish cohort, a larger proportion of recurrent IS events was classified as moderate (19.01% vs 13.84%) or severe (7.92% vs 3.57%) compared with index strokes, indicating greater stroke severity at recurrence (Table 4). This was mainly driven by mild index strokes recurring as strokes of greater severity (moderate or severe).

**Table 3:** Incidence rate and cumulative risk of recurrent ischemic stroke across years of follow-up among patients with non-cardioembolic stroke in England and Denmark

Follow-up	England (n = 52,419)				Denmark (n = 62,501)			
	Person-years	Events, n	IR per 100 person years (95% CI)	Cumulative hazard^a^ per 100 person years (95% CI)	Person-years	Events, n	IR per 100 person years (95% CI)	Cumulative hazard^a^ per 100 person years (95% CI)
Year 1	43,306	3201	7.39 (7.14–7.65)	7.02 (6.78–7.27)	55,996	4458	7.96 (7.73–8.20)	6.92 (6.71–7.14)
Year 2	34,634	1002	2.89 (2.72–3.08)	9.89 (9.60–10.20)	46,655	1591	3.41 (3.25–3.58)	10.31 (10.05–10.59)
Year 3	26,010	612	2.35 (2.17–2.55)	12.24 (11.89–12.60)	37,708	1070	2.84 (2.67–3.01)	13.14 (12.83–13.47)
Year 4	19,149	458	2.39 (2.18–2.62)	14.63 (14.22–15.06)	30,294	787	2.60 (2.42–2.79)	15.73 (15.37–16.11)
Year 5	13,678	260	1.90 (1.68–2.15)	16.53 (16.06–17.01)	23,942	551	2.30 (2.12–2.50)	18.05 (17.64–18.47)

*CI* confidence interval, *IR* incidence rate

^a^Nelson–Aalen cumulative hazard estimate by the end of each period

**Table 4:** Severity of index ischemic stroke and recurrent ischemic stroke in the Danish cohort according to Scandinavian Stroke Scale Score assessed on admission

Severity of index stroke, n (%)	Severity of recurrent IS^a^					
	Mild (score 43–58)	Moderate (score 26–42)	Severe (score 0–25)	Died during admission	Missing	Total
Mild (score 43–58)	3215 (72.12)	722 (16.20)	282 (6.33)	118 (2.65)	121 (2.71)	4458 (80.46)
Moderate (score 26–42)	360 (46.94)	245 (31.94)	106 (13.82)	32 (4.17)	24 (3.13)	767 (13.84)
Severe (score 0–25)	77 (38.89)	59 (29.80)	43 (21.72)	9 (4.55)	10 (5.05)	198 (3.57)
Missing	68 (58.12)	27 (23.08)	8 (6.84)	5 (4.27)	9 (7.69)	117 (2.11)
Total	3720 (67.15)	1053 (19.01)	439 (7.92)	164 (2.96)	164 (2.96)	5540 (100)

*IS* ischemic stroke

^a^Only patients who were identified with recurrent stroke in the Danish Stroke Registry are included here because the Scandinavian Stroke Scale score on admission is only available in this data source. Among the patients with recurrent IS who died during admission, the severity of their stroke on admission was severe in 93 (56.71%), moderate in 31 (18.90%), mild in 35 (21.34%), and missing in 5 (3.05%) patients

In the English cohort, compared to patients classified as fit at the time of index IS, patients with any degree of frailty had a higher risk of recurrence ranging from aHR 1.25 (95% CI, 1.16–1.34) in mild frailty to aHR 1.40 (95% CI 1.22–1.60) in severe frailty (Supplementary Table 5). Overall, associations between baseline risk factors with recurrent IS risk were generally considered weak, with aHRs below 1.5, and were further attenuated in analyses with death as a competing risk. Exceptions that had a higher association with recurrent IS risk included: age at index IS (> 75 years vs < 65 years [reference]), with an aHR of 1.59 (95% CI 1.48–1.72) for the English cohort and 2.07 (95% CI 1.96–2.19) for the Danish cohort; baseline eGFR (< 15 vs ≥ 90 mL/min/1.73 m^2^ [reference]) with an aHR of 1.65 (95% CI 1.20–2.27) for the English cohort (no eGFR data were available for the Danish cohort); and severity of index stroke on admission (Danish data only) with an aHR of 2.12 (95% CI 1.98–2.27) for severe, and 1.51 (95% CI 1.43–1.59) for moderate compared with mild (reference) stroke (Supplementary Table 5).

### Sensitivity analyses

Restricting the follow-up to start on Day 30 after discharge (Supplementary Fig. 3) resulted in somewhat lower first-year IRs per 100 person-years (7.39 [95% CI 7.14–7.65] in the overall English cohort and 5.43 [95% CI 5.20–5.67] in the Day-30–onward cohort). Similarly, in the Danish cohort, the first-year IR was 7.96 (95% CI 7.73–8.20) in the overall cohort and 6.45 (95% CI 6.23–6.68) in the Day-30–onward cohort, with similar trends when stratified by sex, age, and time since index IS (Supplementary Table 6).

At baseline, prevalence of cardiac disorders associated with cardioembolic stroke was < 0.8% in the English cohort and < 1.4% in the Danish cohort (Supplementary Table 7). Of 5857 patients with recurrent IS in the English cohort, 906 (15.5%) were diagnosed with AF during follow-up or within 14 days of being discharged for recurrent IS, compared with 1000 (10.5%) among Danish patients.

## Discussion

In this large, contemporary study of over 114,000 patients with first-ever NCIS in England and Denmark, we found substantial risk of recurrent IS in both countries, despite different data source types. The IR of recurrent IS per 100 person-years was highest in the first year of follow-up (England 7.39; Denmark 7.96) and decreased with time. Despite the IR decline, the 5 year cumulative hazard reached relatively high levels in the English (16.53 per 100 person-years) and Danish (18.05 per 100 person-years) cohorts. Notably, a greater proportion of patients with recurrent IS had a higher degree of frailty (English cohort) and were more frequently recorded as having a moderate or severe recurrent IS (Danish cohort). While age and stroke severity at the index IS were the 2 factors most associated with recurrent IS, most other baseline risk factors demonstrated weak associations.

The accumulation of IS recurrence risk over time has been reported by others [32, 33]. Few studies have examined recurrent IS after an index NCIS specifically. A nationwide Danish study among patients without AF reported 1 and 5 year recurrence risks of 3.9% and 9.6%, respectively. However, a single data source was used to capture recurrence (Stroke Registry) and can explain markedly lower recurrence rates compared with our study [34]. Other reports focus on etiological subgroups (e.g. large-vessel atherosclerosis, small-vessel occlusion) rather than on NCIS overall [11, 35–37]. For example, a German population-based cohort study observed 1 and 5 year cumulative risk of recurrent IS of 7.5% and 20.1%, with strokes of undetermined causes having the highest recurrence risk [35]. A UK study among non-cardioembolic etiological subgroups reported 1 year recurrence rates ranging from 12.3% to 13.4%, and 5 year recurrence rates ranging from 20.0% to 23.4% [37]. A South Korean study including non-cardioembolic subgroups showed 1 year recurrence ranging from 2.0% to 6.6% [36]. While these studies provide insight into stroke recurrence after index NCIS, the data were based on smaller, selected populations, older data, and used different follow-up protocols and outcome statistics, which limit direct comparison.

We found that recurrence rates were largely stable over the study period, except for a decline in the Danish patients aged > 75 years, from 2012 through 2014 to 2018 through 2021. Literature reports on temporal trends of IS recurrence vary, with some studies reporting decreased rates [17, 34, 38], while others, including a recent meta-analysis, show no change [11, 39]. Overall, the varied findings on temporal trends in IS recurrence likely reflect differences in study design, methods of defining recurrence, patient populations, and unique healthcare system variables across countries.

### Strengths and limitations

This study’s key strengths include the large, population-based cohorts from 2 national healthcare systems, which yielded consistent results and enhanced the generalizability of the findings. The use of linked English primary care and hospital data allowed for the analysis of frailty, body mass index, and eGFR, while the use of Danish registry data captured stroke severity scores. The 2 data sources allowed unique comprehensive insight into patient characteristics and outcomes across 2 countries.

However, several methodological limitations should be considered. First, the real-world design may underestimate early in-hospital recurrences as follow-up began after hospital discharge. Conversely, the design likely results in some degree of overestimation of early post-discharge events, as has been shown for re-admissions after intracerebral hemorrhage [40]. This differs from clinical trial settings, whereby recurrent events are adjudicated, ultimately making direct comparison with clinical trials such as CHANCE, POINT, and THALES [19, 20, 41] difficult. Second, differences between cohorts in prevalence of comorbidities and length of hospital stay could reflect variation in data capture and healthcare practices. For example, the higher rate of AF exclusion in the English cohort (38.0%) compared with the Danish cohort (21.0%) could be due to more comprehensive AF screening as well as the fact that AF was only recorded in the primary care setting in the English cohort. In addition, 10.5% (Danish cohort) and 15.5% (English cohort) of NCIS patients with recurrent IS had AF on follow-up, meaning that cardioembolic cases could have been misclassified as NCIS. In the English cohort, of 52,419 patients with NCIS, a total of 2875 had an AF diagnosis recorded within a year of discharge for their NCIS. However, even in the unlikely scenario that all 2875 English cases represented patients who already had paroxysmal AF that was missed due to insufficient cardiac monitoring, this would result in only 5.5% of all English NCIS cases being cardioembolic cases. These differences between cohorts likely have a notable effect on the proportion of patients excluded for cardioembolic stroke and may contribute to small variations in the observed recurrence risk between cohorts. Third, reliance on death certificates for a proportion of outcomes (English cohort: 19.5% vs Danish cohort: 25.1%) may reduce diagnostic accuracy.

An additional consideration is that stroke severity data were only available for patients whose index and recurrent IS were both recorded in the Danish Stroke Registry, which may affect representativeness. Relatedly, in the severity analysis, recurrent IS included both patients discharged alive and those who died during hospitalization, while index IS was restricted to patients discharged alive. This discrepancy could lead to an imbalance, as patients with fatal outcomes at the time of index IS were excluded by design. To address this and provide further data transparency, an additional category for patients who died during admission in the recurrent IS group is included in Table 4. Frailty before stroke is an independent predictor of greater stroke severity in the acute setting [42, 43] and with unscheduled re-admissions in the year following discharge for stroke [44]. As we lacked information on both of these covariates in the same cohort, we could not examine the association between pre-admission frailty and stroke severity upon admission. However, the age- and sex-adjusted HRs for severe frailty in England (2.18 [95% CI 1.96–2.43]) and severe stroke in Denmark (2.22 [95% CI 2.07–2.37]) were nearly identical, suggesting severe frailty and severe stroke are comparably strong predictors of recurrent IS in their respective cohorts. Additional considerations include the potential for residual confounding and other unknown variables and the lack of granular etiology of NCIS defined by cause. Finally, due to lack of available data, we did not assess different index NCIS etiologies, recurrence in different stroke subtype outcomes, or the impact of antiplatelet use during follow-up; the latter 2 analyses are the focus of ongoing research.

It is important to note that stroke management practices in both countries evolved significantly over the study period with an increased use of brain magnetic resonance imaging as part of stroke diagnosis and work-up. This increased the sensitivity for small or posterior circulation infarct detection. These changes, along with increased scan rates more generally, may have contributed to higher detection of index or recurrent IS events in the later years of this study.

## Conclusion

In this large population-based study of over 114,000 participants with first-ever NCIS in England and Denmark, the risk of recurrent IS was highest in the first year, and the cumulative risk of IS recurrence after 5 years was substantial, at approximately 17%. Recurrent strokes were more often classified as severe than the index event, and although most risk factors showed notably weak associations with recurrence risk, age and severity of the index stroke on admission had higher associations with recurrent IS. These findings highlight a continued burden of secondary stroke despite advances in prevention approaches over the last decade and highlight the need for enhanced therapeutic options that reduce the risk of recurrence for people who have suffered a NCIS.

## Supplementary Information

Below is the link to the electronic supplementary material.

Supplementary file1 (DOCX 617 KB)

Supplementary file2 (EPS 1285 KB)

Supplementary file3 (EPS 1354 KB)

Supplementary file4 (EPS 1467 KB)

## Declarations

### Data access, responsibility, and analysis

For the Danish data, Birgit Bjerre Høyer, Sören Möller, and David Gaist conducted the data analyses. For the English data, Antonio Gonzáléz-Pérez and Luis Alberto Garcia Rodriguez conducted the data analyses.

### Data sharing statement

This publication is based on an observational study, which is not within the scope of Bayer’s current data sharing policy (https://clinicaltrials.bayer.com/transparency-policy). As a result, the underlying data from this study are not available for sharing via vivli.org or other data sharing platforms. The public availability of this data is restricted because of participant privacy and data ownership limitations.
