# Supplementary material for: Risk of recurrent ischemic stroke after non-cardioembolic ischemic stroke in England and Denmark
Source: J Neurol. 2026 May 23;273(6):337. doi: 10.1007/s00415-026-13872-4 (PMC13198466; doi:10.1007/s00415-026-13872-4)
Supplement: Supplementary file 1 — Supplementary file1 (DOCX 617 KB) [file 415_2026_13872_MOESM1_ESM.docx]

Supplementary Material

Risk of Recurrent Ischemic Stroke After Non-Cardioembolic Ischemic Stroke in England and Denmark

David Gaist; Antonio Gonzáléz-Pérez; Kristian Tore Jørgensen; Birgit Bjerre Høyer; Sören Möller; Kristina Karlsdotter; Luke Bamber, MSc; Jason Xeni; Deborah Lowe; Mukul Sharma; Luis Alberto Garcia Rodriguez

Corresponding author:

David Gaist, Department of Neurology, Odense University Hospital, Denmark; Email: [dgaist@health.sdu.dk](mailto:dgaist@health.sdu.dk)

[Supplementary Online Content 1](#_Toc220494301)

[Supplementary Methods 3](#_Toc220494302)

[Setting and data sources 3](#_Toc220494303)

[England 3](#_Toc220494304)

[Denmark 4](#_Toc220494305)

[Exclusion of patients from the English and Danish cohorts 5](#_Toc220494306)

[Additional exclusion criteria 5](#_Toc220494307)

[Follow-up 5](#_Toc220494308)

[Outcomes 6](#_Toc220494309)

[Frailty index 6](#_Toc220494310)

[Stroke severity according to the Scandinavian Stroke Scale 7](#_Toc220494311)

[Statistical analysis 7](#_Toc220494312)

[Supplementary Table 1. Diagnosis and covariate codes for studies identified in the Danish data sources 9](#_Toc220494313)

[Supplementary Table 2. Diagnosis codes for study endpoints identified in the English data sources 13](#_Toc220494314)

[Supplementary Table 3. Registry and diagnosis codes of recurrent ischemic stroke recorded among the cohorts by source and country 14](#_Toc220494315)

[Supplementary Table 4. Frailty prior to admission for index ischemic stroke among patients with recurrent ischemic stroke in the English cohort 15](#_Toc220494316)

[Supplementary Table 5. Absolute and relative risk of recurrent ischemic stroke by patient characteristics at the time of the index event, with fully adjusted hazard ratios and subdistribution hazard ratios accounting for competing risk of death 16](#_Toc220494317)

[Supplementary Table 6. Incidence rates of recurrent ischemic stroke among the post-30-day cohorts. A, first year of follow-up. B, all follow-up 24](#_Toc220494318)

[Supplementary Table 7. Cardiac disorders at baseline among patients included in the ischemic stroke cohort in the English (n = 52,419) and Danish (n = 62,501) cohorts 26](#_Toc220494319)

[Supplementary Fig. 1. Ascertainment of patients. A, English (n = 52,419) cohort. B, Danish (n = 62,501) cohort 27](#_Toc220494320)

[Supplementary Fig. 2. Crude cumulative hazard of recurrent IS by sex with 95% CI. A, English (n = 52,419) cohort. B, Danish (n = 62,501) cohort 28](#_Toc220494321)

[Supplementary Fig. 3. Ascertainment of Patients from Day 30 Onward. A, English (n = 47,611) Cohort. B, Danish (n = 58,223) Cohort 29](#_Toc220494322)

[References 30](#_Toc220494323)

# Supplementary Methods

## Setting and data sources

### England

Clinical Practice Research Datalink (CPRD) Aurum is a population-based primary care database from the United Kingdom (UK). Available data include demographics, diagnoses/symptoms, drug exposures, vaccination history, laboratory tests, and referrals to hospital and specialist care. CPRD Aurum has been intensively used in research [1, 2] and includes around 13 million active patients who represent around 20% of the total UK population and a similar proportion of general practices [3]. Therefore, data from CPRD Aurum are widely representative of the UK general population and of England in terms of geographical spread, deprivation, age, and sex. Historic and current patients acceptable for research add up to a total of 41 million patients. Only data from practices that have consented to participate in the CPRD linkage scheme and have provided patient-level information were used, which includes 90% of all practices [3]. The linked subset covers approximately 50–60% of CPRD Aurum practices. Study endpoints were identified from linked hospitalizations recorded within the Hospital Episode Statistics (HES) Admitted Patient Care (APC) dataset [4]. Deaths are routinely recorded in CPRD, with more than 98% of deaths correctly recorded, although the exact date of death tends to be slightly delayed compared with Office of National Statistics (ONS) data [5, 6]. Thus, ONS-linked death data were obtained as source of information on mortality to obtain exact dates and comprehensive recording on cause of death. Consequently, only English practices with linkage to HES and ONS were used in this study.

### Denmark

All residents of Denmark have a unique and permanent personal identification number [7], which is recorded in the Danish registries used in this study. We used this 10-digit identification number to link data across registries at the level of the individual, as in previous studies [8].

The Danish Stroke Registry (Stroke Registry) contains prospectively collected data on all patients hospitalized in Denmark, primarily from all the stroke units in the country. In the most recent study of the validity of diagnoses in the Stroke Registry, sensitivity was reported to be 97% [9].

The Danish National Patient Registry (Patient Registry) has recorded information on all hospital contacts (admissions since 1977, outpatient contacts since 1995) in Denmark [10]. Data recorded include diagnoses according to the International Classification of Diseases (ICD; version 8 until 1993, version 10 since 1994), contact dates, and codes for procedures performed.

The Danish National Prescription Registry (Prescription Registry) holds data on all prescriptions redeemed at community pharmacies in Denmark since 1995 [11]. For each prescription, the date of dispensing and a full account of the dispensed product, including the anatomical therapeutic chemical code and a unique package-specific code are recorded [11, 12]. The indication and prescribed dose are not available in the Prescription Registry [12].

The Danish Register of Causes of Death, dating back to 1875, has held computerized individual records since 1970, with cause(s) of death being classified according to the rule of the World Health Organization. Since 1994, this is by ICD-10 codes [13].

The Danish Civil Registration System is the backbone of administrative systems in Denmark and has also been used extensively for research [7], as it is continuously updated with regard to individuals’ migration and vital status, including date of death.

## Exclusion of patients from the English and Danish cohorts

Patients with recorded use of oral anticoagulants (OACs) in the 90 days leading up to the index ischemic stroke (IS) admission were excluded unless they fulfilled one of the following conditions:

1. A diagnosis of venous thromboembolism (deep vein thrombosis or pulmonary embolism) at any time before and up to 1 day before the date of index IS hospitalization, or
2. A record of hip/knee surgery from 180 days to 1 day before the index IS hospitalization.

These two exceptions were introduced as they represent non-atrial fibrillation indications for OAC use in England and Denmark.

### Additional exclusion criteria

Additional exclusions included patients with a diagnosis of IS predating the index IS hospitalization and those who died during admission for the index event (**Supplementary Fig. 1**).

## Follow-up

Follow-up began the day after hospital discharge for the index IS hospitalization and continued until the earliest of either the recurrent IS or the end of the study period (March 2021 for England and August 2022 for Denmark). Follow-up also ended at the last date of data collection in that practice for the English cohort or emigration for the Danish cohort. Thus, for patients who had more than one recurrent IS in the study period, follow-up ended at the first recurrent IS event. Atrial fibrillation recorded during follow-up was not censored (apart from for the first 15 days, see above), nor was initiation of OACs.

## Outcomes

The study outcome was recurrent IS, defined as the first (chronological) hospitalization with a primary diagnosis code of IS (ICD-10: I63/I64) recorded during follow-up as captured through the HES APC (England), Stroke Registry (Denmark), Patient Registry (Denmark), or Death Registry (Denmark, England) (see **Supplementary Table 3**). Ascertainment of recurrent IS was conducted in a descending hierarchical order (HES APC over Death Registry in England; Stroke Registry over Patient Registry over Death Registry in Denmark).

Outcomes in the Danish cohort were ascertained exclusively using the Stroke Registry for days 1 to 14 of follow-up. Formal validation studies of the registered data have not been conducted; however, the overall quality is likely high, as detailed instructions and explicit data definitions are provided by the Stroke Registry, and data-recording practices are reviewed and discussed by representatives from the reporting hospital departments during annual audits [14]. Similarly, outcomes in the English cohort were solely ascertained using HES APC for days 1 to 14 of follow-up. Among patients with recurrent IS in both cohorts, the number who had received an atrial fibrillation diagnosis during follow-up or up to 15 days after their recurrent IS was calculated.

### Frailty index

We measured frailty by estimating the electronic frailty index (eFI), which identifies the presence of 36 specified deficits based on primary care electronic health records [15]. Thus, data extracted from Aurum were used to calculate the eFI score as a cumulative proportion of those 36 deficits that had been recorded prior to admission for the index IS. People were divided into categories (fit, mild, moderate, and severe frailty) based on these scores. These categories have been shown to be predictive of subsequent mortality and emergency hospital and nursing home admission [15]. Furthermore, the eFI has been recently used to identify longitudinal trajectories of frailty using data from Aurum [16].

We also calculated the eFI among patients with recurrent IS, as outlined above and using information from Aurum up to the day before re-admission. However, we assigned all patients with recurrent IS the eFI item “cerebrovascular disease” regardless of Aurum status, as we were cognizant of their re-admission for recurrent IS.

### Stroke severity according to the Scandinavian Stroke Scale

For the index IS and recurrent IS, the median and interquartile range of the Scandinavian Stroke Scale (SSS) score were calculated; scores were grouped into mild (43–58), moderate (26–42), and severe (0–25), corresponding closely with categories of the National Institutes of Health Stroke Scale (mild: 0–5; moderate: 6–14; and severe: 15–31) [17].

## Statistical analysis

The full model (adjusted hazard ratio [HR]) estimating the risk of recurrent IS contained additional adjustment for the following factors: smoking (current, former, never, missing), hypertension, diabetes, hyperlipidemia, peripheral artery disease, ischemic heart disease, congestive heart failure, prior transient ischemic attack (TIA), major bleeding event, venous thromboembolism, dementia, chronic kidney disease, chronic hepatic disease, cancer (except skin cancer), chronic obstructive pulmonary disease, disorders indicative of high alcohol use, and use of medications at the time of index IS admission (separate variables for each of the following: antiplatelets, OACs, statins, thiazides and other non-loop diuretics, loop diuretics, beta-blockers, calcium channel blockers, angiotensin-converting enzyme inhibitors and angiotensin-receptor blockers, non-steroidal anti-inflammatory drugs, selective serotonin reuptake inhibitors, and proton pump inhibitors).

**Supplementary Table 1**. Diagnosis and covariate codes for studies identified in the Danish data sources

|  | **Diagnosis code (ICD-10)/procedure code from Danish National Patient Registry (unless otherwise specified)** | **Drug (ATC) code** |
| --- | --- | --- |
| IS event (index or recurrent) | | |
| IS (index) | I63 (in the Danish Stroke Registry) | NA |
| IS (recurrent) | I63, I64 (in the Danish Stroke Registry, Patient Registry, or Death Registry) | NA |
| Diagnosis code used to exclude patients with atrial fibrillation | | |
| Atrial fibrillation | I48 | NA |
| Diagnosis/procedure codes to identify non-atrial fibrillation use of OAC before admission for index stroke | | |
| Venous thromboembolism (DVT/PE) | I26, I801, I802, I803, I808, I809 | NA |
| Arthroplasty surgery (hip or knee) | Operation procedure codes: KNFB, KNFC, KNGB, KNGC | NA |
| Covariates – disorders^a^  *For diagnosis codes at baseline inpatient or outpatient, primary or secondary position (unless otherwise specified)* | | |
| Hypertension | I10–I15 | C03A, C08CA, C08DB01, C09A, C09B, C09C, C09D |
| Prior IS (i.e., before entry stroke) | I63, I64 (in the Danish Stroke Registry or Danish National Patient Registry) | NA |
| Prior transient ischemic attack (i.e., before index stroke) | G45 (except G454) | NA |
| Intracranial hemorrhage (ICH, SAH, SDH) Diagnosis codes with primary position inpatient and outpatient | ICH – I61 (in the Danish Stroke Registry or Patient Registry) SAH – I60 SDH – I62, S064, S065, S066 | NA |
| Ischemic heart disease | I20, I21, I22, I23, I24, I25 | NA |
| Peripheral arterial disease | I702, I739 | NA |
| Venous thromboembolism (DVT/PE) | I26, I801, I802, I803, I808, I809 | NA |
| Diabetes | E10–E14 | A10 (excluding drugs used for weight loss identified based on trade names) |
| Hyperlipidemia | E78 | NA |
| Chronic kidney failure | N18 (excluding N181), N19, Z992, Z940 | NA |
| Chronic hepatic diseases | K71–K77 | NA |
| Congestive heart failure | I110, I130, I132, I420, I426, I427, I428, I429, I50 | NA |
| Dementia | F00, F01–F03, F05.1, G30 | NA |
| Cancer | C00–C97 (excluding C44) | NA |
| Disorders/events or drug use indicative of alcohol misuse | E244, F10, G312, G621, G721, I426, K292, K70, K860, T510, T519, Z502, Z714, Z721 | N07BB |
| Chronic obstructive pulmonary disorder | J42, J43, J44 | NA |
| Gastrointestinal bleed (upper, lower, or unspecified) Diagnosis codes with primary position inpatient and outpatient | I850, K228F, K250, K252, K254, K256, K260, K262, K264, K266, K298A, K270, K272, K274, K276, K280, K282, K284, K286, K290, K625, K661, K920, K921, K922, K638B, K638C | NA |
| Major bleed event Diagnosis codes with primary position inpatient and outpatient | Intracranial hemorrhage See above Gastrointestinal bleed See above Urogenital bleed N020–N029, N421, N836, N857, N897, N920–N925, N930, N938, N939, N950, R319, S314 | NA |
| Covariates – use of medications^b^ |  |  |
| Antiplatelet drugs |  |  |
| Aspirin – low dose | NA | B01AC06 – acetylsalicylic acid (75 mg, 100 mg, or 150 mg per tablet) B01AC30 – acetylsalicylic acid (25 mg per tablet) in combination with dipyridamole |
| Clopidogrel | NA | B01AC04 (75 mg per tablet) |
| Dipyridamole | NA | B01AC07 (100 mg or 200 mg per tablet) |
| Other ADP receptor blockers | NA | B01AC22 – prasugrel (5 mg or 10 mg per tablet) B01AC24 – ticagrelor (60 mg or 90 mg per tablet) |
| Anticoagulant drugs |  |  |
| Vitamin K antagonists | NA | B01AA |
| Direct oral anticoagulants | NA |  |
| Dabigatran | NA | B01AE07 |
| Rivaroxaban | NA | B01AF01 |
| Apixaban | NA | B01AF02 |
| Edoxaban | NA | B01AF03 |
| Drugs with antihypertensive effects^c^ |  |  |
| Thiazides and other non-loop diuretics | NA | C03A, C03D, C03E |
| Loop diuretics | NA | C03C |
| Beta-blockers | NA | C07 |
| Calcium channel blockers | NA | C08 |
| ACE inhibitors and angiotensin II receptor blockers (plain or in combinations) | NA | C09A, C09B, C09C, C09D |
| Digoxin | NA | C01AA05 |
| Statins | NA | C10AA |
| SSRIs | NA | N06AB |
| NSAIDs | NA | M01A (including Cox2 inhibitors), excluding M01AX |
| PPIs | NA | A02BC |
| Codes used in sensitivity analysis to identify cardiac disorders at baseline among patients included in the cohort | | |
| Mechanical heart valve (independent of atrial fibrillation codes) | Procedure codes (since 1996; no specific codes available before 1996):  KFKD00, KFMD00 KFGE00 KFJF00 | NA |
| Patent foramen ovale (undergone percutaneous transluminal closure) | ICD-10: Q211C  & Procedure code: KFFC42 within 180 days of IS diagnosis | NA |
| Cardiomyopathy | I255, I42, I43 | NA |
| Endocarditis | I33, I389, I398 | NA |
| Left ventricular thrombus | I513 | NA |
| Atrial myxoma | D151=benign cardiac tumor; no specific ICD code for myxoma | NA |
| Cardiac tumors, malignant or benign | C381, C388, D151 | NA |
| Aortic surgery within 30 days before the index stroke discharge date | Procedure codes: Transcatheter aortic valve implantation: KFMD12 Aorta coronary artery bypass: KFNC | NA |

*ACE* angiotensin-converting enzyme; *ADP* adenosine diphosphate; *ATC* anatomical therapeutic chemical; *DVT* deep vein thrombosis; *ICD-10* International Classification of Diseases, Tenth Revision; *ICH* intracranial hemorrhage; *IS* ischemic stroke; *NA* not applicable; *NSAID* non-steroidal anti-inflammatory drug; *OAC* oral anticoagulant; *PE* pulmonary embolism; *PPI* proton pump inhibitor; *SAH* subarachnoid hemorrhage; *SDH* subdural hemorrhage; *SSRI* selective serotonin reuptake inhibitor.
^a^ICD codes (or where applicable, ATC codes) using all available data up to date of index stroke. ^b^Current use was defined as prescription supply covering the date of entry stroke or ending within 90 days of entry stroke. ^c^Indication for drug use not available; some drugs may have been used for indications other than hypertension. Note that “ever use” of some drugs is included in the definition of the hypertension covariate (i.e., C03A, C08CA, C08DB01, C09A, C09B, C09C, C09D).

**Supplementary Table 2**. Diagnosis codes for study endpoints identified in the English data sources

|  | **Diagnosis code (ICD-10) in primary position in HES APC or Death Registry (unless otherwise specified)** |
| --- | --- |
| Ischemic stroke event (index or recurrent) | |
| Ischemic stroke (index) | I63 (in HES APC only) |
| Ischemic stroke (recurrent) | I63, I64 |
| Other study endpoints | |
| Intracranial hemorrhage | I60, I61, I62, S064, S065, S066 |
| Gastrointestinal bleed (upper, lower, or unspecified) | I850, K228F, K250, K252, K254, K256, K260, K262, K264, K266, K298A, K270, K272, K274, K276, K280, K282, K284, K286, K290, K625, K661, K920, K921, K922, K638B, K638C |
| Major bleed event | Intracranial hemorrhage See above Gastrointestinal bleed See above Urogenital bleed N020–N029, N421, N836, N857, N897, N920–N925, N930, N938, N939, N950, R319, S314 |

*APC* Admitted Patient Care; *HES* Hospital Episode Statistics; *ICD-10* International Classification of Diseases, Tenth Revision.

Supplementary Table 3. Registry and diagnosis codes of recurrent ischemic stroke recorded among the cohorts by source and country

| **Source, n (%)** | **England (n = 5857)** | **Denmark (n = 9019)** |
| --- | --- | --- |
| Stroke Registry (Denmark)/HES APC (England) | 4717 (80.5) | 5540 (58.4) |
| I63^a^ | 4398 (75.1) | 5464 (57.6) |
| I64^b^ | 319 (5.4) | 76 (0.8) |
| Patient Registry (Denmark) | – | 1563 (16.5) |
| I63^a^ | – | 947 (10.0) |
| I64^b^ | – | 616 (6.5) |
| Death Registry (Denmark and England) | 1140 (19.5) | 2386 (25.1) |
| I63^a^ | 120 (2.0) | 334 (3.5) |
| I64^b^ | 1020 (17.4) | 2052 (21.6) |

*APC* Admitted Patient Care; *HES* Hospital Episode Statistics; *ICD-10* International Classification of Diseases Tenth Revision; *IS* ischemic stroke.
Note: Ascertainment of recurrent IS was conducted in a descending hierarchical order (HES APC over Death Registry in England; Stroke Registry over Patient Registry over Death Registry in Denmark). ^a^ICD-10 code for IS. ^b^ICD-10 code for stroke, unspecified.

Supplementary Table 4. Frailty prior to admission for index ischemic stroke among patients with recurrent ischemic stroke in the English cohort

|  | **Frailty^a^ at admission for recurrent IS** | | | | |
| --- | --- | --- | --- | --- | --- |
| Frailty^a^ at admission for index IS, n (%) | Fit | Mild frailty | Moderate frailty | Severe frailty | Total |
| Fit | 1189 (55.69) | 866 (40.56) | 73 (3.42) | 7 (0.33) | 2135 (36.45) |
| Mild frailty | 5 (0.24) | 1222 (58.05) | 798 (37.91) | 80 (3.80) | 2105 (35.94) |
| Moderate frailty | 0 (0.00) | 0 (0.00) | 758 (63.54) | 435 (36.46) | 1193 (20.37) |
| Severe frailty | 0 (0.00) | 0 (0.00) | 2 (0.47) | 422 (99.53) | 424 (7.24) |
| Total | 1194 (20.39) | 2088 (35.65) | 1631 (27.85) | 944 (16.12) | 5857 (100.00) |

*IS* ischemic stroke. ^a^Electronic frailty score estimated from primary care physician data up to the day before the date of admission (index IS) and date of re-admission (recurrent IS), classified as: fit (≤0.12), mild frailty (0.13–0.24), moderate frailty (0.25–0.36), or severe frailty (≥0.36).

Supplementary Table 5. Absolute and relative risk of recurrent ischemic stroke by patient characteristics at the time of the index event, with fully adjusted hazard ratios and subdistribution hazard ratios accounting for competing risk of death

|  | **England (n = 52,419)** | | | | **Denmark (n = 62,501)** | | | |
| --- | --- | --- | --- | --- | --- | --- | --- | --- |
|  | **IR per 100 person-years** | **HR^a^  (95% CI)** | **aHR^b^ (95% CI)** | **sHR^c^  (95% CI)** | **IR per 100 person-years** | **HR^a^  (95% CI)** | **aHR^b^  (95% CI)** | **sHR^c^  (95% CI)** |
| Sex |  |  |  |  |  |  |  |  |
| Males | 3.5 | 1 (ref) | 1 (ref) | 1 (ref) | 3.8 | 1 (ref) | 1 (ref) | 1 (ref) |
| Females | 4.0 | 1.03 (0.98–1.09) | 1.05 (0.99–1.11) | 1.05 (1.00–1.11) | 4.0 | 0.94 (0.90–0.98) | 0.94 (0.90–0.98) | 0.95 (0.91–0.99) |
| Age categories, years |  |  |  |  |  |  |  |  |
| <65 | 2.5 | 1 (ref) | 1 (ref) | 1 (ref) | 2.4 | 1 (ref) | 1 (ref) | 1 (ref) |
| 65–75 | 3.3 | 1.31 (1.22–1.40) | 1.17 (1.09–1.27) | 1.14 (1.06–1.23) | 3.5 | 1.41 (1.34–1.49) | 1.33 (1.25–1.40) | 1.26 (1.19–1.34) |
| >75 | 5.5 | 1.94 (1.82–2.07) | 1.59 (1.48–1.72) | 1.43 (1.33–1.54) | 6.3 | 2.33 (2.21–2.45) | 2.07 (1.96–2.19) | 1.72 (1.63–1.82) |
| Townsend deprivation index (quintiles) |  |  |  |  |  |  |  |  |
| Least deprived | 3.5 | 1 (ref) | 1 (ref) | 1 (ref) | NA | NA | NA | NA |
| 20–40% | 3.7 | 1.07 (0.99–1.16) | 1.04 (0.96–1.13) | 1.05 (0.97–1.14) | NA | NA | NA | NA |
| 40–60% | 3.6 | 1.07 (0.98–1.16) | 1.02 (0.94–1.11) | 1.02 (0.94–1.11) | NA | NA | NA | NA |
| 60–80% | 3.8 | 1.15 (1.06–1.25) | 1.07 (0.99–1.16) | 1.07 (0.98–1.16) | NA | NA | NA | NA |
| Most deprived | 4.1 | 1.31 (1.21–1.42) | 1.17 (1.07–1.27) | 1.16 (1.07–1.26) | NA | NA | NA | NA |
| Unknown | 4.2 | 1.22 (0.55–2.73) | 1.26 (0.56–2.80) | 1.24 (0.55–2.80) | NA | NA | NA | NA |
| BMI, kg/m^2^ |  |  |  |  |  |  |  |  |
| <20 | 5.1 | 1.19 (1.06–1.34) | 1.21 (1.08–1.37) | 1.14 (1.01–1.28) | NA | NA | NA | NA |
| 20–24.9 | 3.9 | 1 (ref) | 1 (ref) | 1 (ref) | NA | NA | NA | NA |
| 25–29.9 | 3.4 | 0.92 (0.86–0.99) | 0.89 (0.83–0.96) | 0.92 (0.86–0.99) | NA | NA | NA | NA |
| ≥30 | 3.5 | 1.03 (0.95–1.11) | 0.91 (0.84–0.98) | 0.95 (0.88–1.02) | NA | NA | NA | NA |
| Unknown | 4.4 | 1.13 (1.04–1.22) | 1.17 (1.08–1.27) | 1.13 (1.04–1.23) | NA | NA | NA | NA |
| Smoking status |  |  |  |  |  |  |  |  |
| Current | 3.3 | 1.09 (1.01–1.18) | 1.08 (0.99–1.17) | 1.05 (0.97–1.14) | 3.5 | 1.17 (1.11–1.23) | 1.13 (1.07–1.19) | 1.08 (1.02–1.14) |
| Past | 4.0 | 1.05 (0.98–1.11) | 0.98 (0.92–1.05) | 0.98 (0.92–1.04) | 3.9 | 1.01 (0.95–1.06) | 0.95 (0.90–1.01) | 0.94 (0.89–0.99) |
| Never | 3.8 | 1 (ref) | 1 (ref) | 1 (ref) | 3.7 | 1 (ref) | 1 (ref) | 1 (ref) |
| Missing | 3.7 | 1.10 (1.00–1.22) | 1.23 (1.11–1.37) | 1.20 (1.09–1.33) | 5.6 | 1.35 (1.26–1.44) | 1.28 (1.20–1.37) | 1.14 (1.06–1.22) |
| Number of hospitalizations in the previous year, any diagnosis, No. |  |  |  |  |  |  |  |  |
| 0 | 3.3 | 1 (ref) | 1 (ref) | 1 (ref) | 3.5 | 1 (ref) | 1 (ref) | 1 (ref) |
| 1 | 4.3 | 1.21 (1.14–1.30) | 1.09 (1.02–1.17) | 1.07 (1.00–1.14) | 4.8 | 1.28 (1.22–1.35) | 1.16 (1.10–1.23) | 1.11 (1.05–1.17) |
| 2 | 5.1 | 1.36 (1.24–1.49) | 1.15 (1.04–1.26) | 1.11 (1.01–1.22) | 5.6 | 1.44 (1.32–1.56) | 1.22 (1.12–1.33) | 1.11 (1.02–1.22) |
| ≥3 | 6.1 | 1.57 (1.43–1.72) | 1.23 (1.12–1.36) | 1.09 (0.99–1.21) | 6.1 | 1.55 (1.41–1.70) | 1.23 (1.11–1.36) | 1.03 (0.93–1.14) |
| Number of outpatient visits in previous year, any diagnosis, No. |  |  |  |  |  |  |  |  |
| 0 | 3.0 | 1 (ref) | 1 (ref) | 1 (ref) | NA | NA | NA | NA |
| 1–2 | 3.6 | 1.08 (1.00–1.16) | 1.00 (0.93–1.08) | 1.00 (0.93–1.08) | NA | NA | NA | NA |
| 3–5 | 3.9 | 1.13 (1.04–1.21) | 1.00 (0.92–1.08) | 1.00 (0.92–1.08) | NA | NA | NA | NA |
| ≥6 | 5.1 | 1.44 (1.35–1.53) | 1.17 (1.09–1.26) | 1.13 (1.05–1.21) | NA | NA | NA | NA |
| Number of primary care visits in previous year, any diagnosis, No. |  |  |  |  |  |  |  |  |
| 0–4 | 2.4 | 1 (ref) | 1 (ref) | 1 (ref) | NA | NA | NA | NA |
| 5–9 | 2.9 | 1.11 (1.00–1.23) | 1.04 (0.94–1.15) | 1.03 (0.93–1.14) | NA | NA | NA | NA |
| 10–19 | 3.8 | 1.38 (1.27–1.51) | 1.19 (1.08–1.30) | 1.17 (1.07–1.29) | NA | NA | NA | NA |
| ≥20 | 4.9 | 1.67 (1.53–1.82) | 1.26 (1.15–1.39) | 1.21 (1.10–1.34) | NA | NA | NA | NA |
| Frailty^d^ |  |  |  |  |  |  |  |  |
| Fit | 2.6 | 1 (ref) | 1 (ref) | 1 (ref) | NA | NA | NA | NA |
| Mild | 4.3 | 1.45 (1.36–1.54) | 1.25 (1.16–1.34) | 1.22 (1.13–1.31) | NA | NA | NA | NA |
| Moderate | 6.2 | 1.84 (1.71–1.99) | 1.37 (1.25–1.51) | 1.30 (1.18–1.43) | NA | NA | NA | NA |
| Severe | 8.4 | 2.18 (1.96–2.43) | 1.40 (1.22–1.60) | 1.29 (1.12–1.48) | NA | NA | NA | NA |
| Severity of entry stroke on admission^e^ |  |  |  |  |  |  |  |  |
| Mild (score 43–58) | NA | NA | NA | NA | 3.26 | 1 (ref) | 1 (ref) | 1 (ref) |
| Moderate (score 26–42) | NA | NA | NA | NA | 6.05 | 1.59 (1.50–1.67) | 1.51 (1.43–1.59) | 1.35 (1.28–1.42) |
| Severe (score 0–25) | NA | NA | NA | NA | 8.17 | 2.22 (2.07–2.37) | 2.12 (1.98–2.27) | 1.61 (1.50–1.73) |
| Missing | NA | NA | NA | NA | 4.03 | 1.28 (1.12–1.46) | 1.19 (1.04–1.35) | 1.12 (0.98–1.28) |
| Comorbidity at the time of the index stroke^f^ |  |  |  |  |  |  |  |  |
| Hypertension | 4.6 | 1.43 (1.35–1.51) | 1.21 (1.12–1.30) | 1.17 (1.09–1.26) | 4.5 | 1.30 (1.24–1.37) | 1.17 (1.11–1.24) | 1.16 (1.09–1.22) |
| Diabetes | 5.4 | 1.52 (1.44–1.61) | 1.35 (1.27–1.44) | 1.31 (1.23–1.39) | 5.1 | 1.31 (1.24–1.38) | 1.16 (1.10–1.23) | 1.12 (1.06–1.18) |
| Hyperlipidemia | 4.6 | 1.22 (1.16–1.29) | 0.97 (0.91–1.03) | 0.98 (0.92–1.04) | 3.7 | 0.98 (0.94–1.02) | 0.88 (0.84–0.92) | 0.93 (0.89–0.98) |
| Peripheral artery disease | 6.2 | 1.51 (1.38–1.66) | 1.17 (1.06–1.29) | 1.11 (1.01–1.23) | 7.2 | 1.62 (1.50–1.74) | 1.33 (1.23–1.44) | 1.23 (1.13–1.33) |
| Ischemic heart disease | 5.5 | 1.46 (1.38–1.55) | 1.12 (1.04–1.20) | 1.11 (1.03–1.19) | 5.4 | 1.30 (1.24–1.37) | 1.08 (1.02–1.15) | 1.07 (1.00–1.13) |
| Congestive heart failure | 6.2 | 1.37 (1.23–1.52) | 0.97 (0.87–1.09) | 0.91 (0.81–1.02) | 6.3 | 1.37 (1.25–1.49) | 1.06 (0.96–1.17) | 0.97 (0.88–1.07) |
| Transient ischemic attack | 5.8 | 1.43 (1.32–1.54) | 1.23 (1.13–1.33) | 1.22 (1.13–1.33) | 5.3 | 1.31 (1.23–1.40) | 1.21 (1.13–1.29) | 1.22 (1.14–1.31) |
| Major bleeding event (intracranial, gastrointestinal, or genitourinary) | 4.2 | 1.11 (1.05–1.18) | 1.01 (0.95–1.07) | 1.01 (0.96–1.07) | 4.1 | 1.08 (1.02–1.14) | 1.00 (0.94–1.06) | 0.99 (0.94–1.05) |
| Intracranial hemorrhage (ICH, SAH, or SDH) | 4.9 | 1.22 (1.03–1.44) | NA | NA | 5.3 | 1.27 (1.12–1.44) | NA | NA |
| Gastrointestinal bleed (upper, lower, or unspecified) | 4.4 | 1.12 (1.05–1.19) | NA | NA | 5.4 | 1.25 (1.15–1.36) | NA | NA |
| Venous thromboembolism | 5.0 | 1.22 (1.11–1.35) | 1.12 (1.01–1.24) | 1.11 (1.00–1.23) | 5.5 | 1.25 (1.16–1.36) | 1.13 (1.03–1.23) | 1.08 (0.99–1.18) |
| Dementia | 8.5 | 1.43 (1.28–1.60) | 1.29 (1.15–1.44) | 1.07 (0.95–1.20) | 8.4 | 1.46 (1.32–1.61) | 1.32 (1.19–1.46) | 1.00 (0.90–1.11) |
| CKD^g^ | 6.2 | 1.40 (1.31–1.49) | 1.18 (1.10–1.26) | 1.14 (1.06–1.21) | 7.3 | 1.48 (1.32–1.65) | 1.20 (1.07–1.34) | 1.08 (0.96–1.21) |
| Chronic hepatic disease | 4.3 | 1.14 (1.03–1.27) | 1.00 (0.90–1.11) | 0.97 (0.87–1.09) | 4.6 | 1.19 (1.02–1.40) | 1.04 (0.89–1.23) | 1.00 (0.85–1.18) |
| Cancer | 4.8 | 1.11 (1.04–1.19) | 1.08 (1.01–1.15) | 0.99 (0.92–1.06) | 5.0 | 1.06 (1.01–1.12) | 1.04 (0.98–1.09) | 0.92 (0.87–0.97) |
| COPD | 4.8 | 1.19 (1.10–1.28) | 1.06 (0.98–1.15) | 1.02 (0.94–1.10) | 5.1 | 1.15 (1.07–1.24) | 1.01 (0.93–1.08) | 0.93 (0.86–1.00) |
| Disorders indicative of high alcohol use | 3.9 | 1.19 (1.09–1.31) | 1.11 (1.01–1.22) | 1.09 (0.99–1.20) | 4.0 | 1.25 (1.17–1.34) | 1.13 (1.06–1.22) | 1.09 (1.02–1.17) |
| Baseline eGFR (mL/min/ 1.73 m^2^)^h^ |  |  |  |  |  |  |  |  |
| ≥90 | 3.0 | 1 (ref) | 1 (ref) | 1 (ref) | NA | NA | NA | NA |
| 60–89 | 3.8 | 0.92 (0.82–1.03) | 0.92 (0.82–1.03) | 0.95 (0.85–1.06) | NA | NA | NA | NA |
| 45–59 | 4.8 | 1.02 (0.90–1.16) | 0.95 (0.83–1.09) | 0.98 (0.85–1.12) | NA | NA | NA | NA |
| 30–44 | 6.9 | 1.32 (1.15–1.53) | 1.12 (0.96–1.30) | 1.11 (0.95–1.29) | NA | NA | NA | NA |
| 15–29 | 9.3 | 1.66 (1.37–2.02) | 1.23 (1.00–1.52) | 1.14 (0.92–1.40) | NA | NA | NA | NA |
| <15 | 12.1 | 2.41 (1.78–3.28) | 1.65 (1.20–2.27) | 1.35 (0.98–1.88) | NA | NA | NA | NA |
| Unknown | 3.3 | 0.89 (0.80–0.99) | 0.98 (0.88–1.09) | 1.00 (0.90–1.11) | NA | NA | NA | NA |
| Use of medications at the time of the index stroke^i^ |  |  |  |  |  |  |  |  |
| Oral anticoagulants | 6.1 | 1.5 (1.18–1.91) | 1.33 (1.03–1.73) | 1.30 (1.00–1.69) | 6.3 | 1.33 (1.10–1.62) | 1.11 (0.99–1.26) | 1.09 (0.96–1.23) |
| Antiplatelets | 5.5 | 1.54 (1.46–1.63) | 1.26 (1.18–1.36) | 1.26 (1.17–1.35) | 5.8 | 1.46 (1.40–1.53) | 1.24 (1.18–1.29) | 1.21 (1.16–1.27) |
| Statins | 4.5 | 1.26 (1.20–1.33) | 0.91 (0.85–0.97) | 0.92 (0.86–0.99) | 4.6 | 1.16 (1.11–1.22) | 0.95 (0.91–1.00) | 0.98 (0.94–1.03) |
| Thiazides and other non-loop diuretics | 4.0 | 1.01 (0.94–1.09) | 0.93 (0.86–1.00) | 0.94 (0.87–1.01) | 4.8 | 1.13 (1.07–1.19) | 1.03 (0.99–1.08) | 1.04 (0.99–1.09) |
| Loop diuretics | 6.4 | 1.43 (1.31–1.55) | 1.14 (1.04–1.24) | 1.06 (0.97–1.16) | 6.5 | 1.36 (1.27–1.45) | 1.09 (1.02–1.15) | 0.98 (0.92–1.05) |
| Beta-blockers | 5.0 | 1.31 (1.24–1.39) | 1.07 (1.00–1.15) | 1.08 (1.01–1.16) | 5.1 | 1.24 (1.18–1.30) | 1.05 (1.00–1.10) | 1.04 (0.99–1.10) |
| Calcium channel blockers | 4.5 | 1.18 (1.11–1.24) | 1.02 (0.96–1.09) | 1.03 (0.97–1.10) | 4.8 | 1.15 (1.09–1.20) | 1.01 (0.97–1.06) | 1.01 (0.97–1.06) |
| ACE inhibitors and angiotensin-receptor blockers | 4.4 | 1.22 (1.16–1.29) | 0.96 (0.91–1.03) | 1.00 (0.94–1.06) | 4.5 | 1.12 (1.07–1.17) | 0.96 (0.92–1.00) | 0.97 (0.93–1.01) |
| NSAIDs | 3.0 | 0.87 (0.79–0.96) | 0.90 (0.81–1.00) | 0.92 (0.83–1.02) | 3.7 | 1.03 (0.96–1.10) | 1.00 (0.97–1.03) | 1.01 (0.98–1.05) |
| SSRIs | 4.3 | 1.19 (1.09–1.29) | 1.06 (0.97–1.16) | 1.04 (0.96–1.14) | 5.2 | 1.30 (1.21–1.40) | 1.10 (1.03–1.16) | 1.07 (1.01–1.13) |
| PPIs | 4.5 | 1.19 (1.13–1.26) | 1.03 (0.97–1.09) | 1.01 (0.95–1.07) | 5.0 | 1.19 (1.14–1.25) | 1.02 (0.98–1.06) | 1.00 (0.96–1.04) |

*ACE* angiotensin-converting enzyme; *aHR* adjusted hazard ratio; *APC* Admitted Patient Care; *BMI* body mass index; *CI* confidence interval; *CKD* chronic kidney disease; *COPD* chronic obstructive pulmonary disease; *CPRD* Clinical Practice Research Datalink; *eGFR* estimated glomerular filtration rate; *HES* Healthcare Episode Statistics; *HR* hazard ratio; *ICH* intracerebral hemorrhage; *IR* incidence rate; *NA* not applicable; *NSAID* non-steroidal anti-inflammatory drug; *PPI* proton pump inhibitor; *ref* reference; *SAH* subarachnoid hemorrhage; *SDH* subdural hemorrhage; *sHR* subdistribution hazard ratio; *SSRI* selective serotonin reuptake inhibitor. ^a^HR and 95% CI estimates adjusted by age and sex using Cox proportional hazards models.  ^b^HR and 95% CI estimates adjusted by age, sex, smoking, comorbidities listed above, and use of medications listed above using Cox proportional hazards models. ^c^sHR and 95% CI estimates adjusted by age, sex, smoking, comorbidities listed above, and use of medications listed above using the Fine & Gray regression model with death as the competing risk. ^d^Based on electronic frailty index score calculated using all available information in primary care electronic health records up to the day before admission for index ischemic stroke. Based on this score, frailty was classified into fit (≤0.12), mild (0.13–0.24), moderate (0.25–0.36), and severe (≥0.36). ^e^Measured using the Scandinavian Stroke Scale score as recorded in the Stroke Registry. ^f^Any time before the date of the index stroke. ^g^In the English cohort, based on recorded diagnoses (primary care in CPRD Aurum or hospital admissions in HES APC) compatible with Kidney Disease: Improving Global Outcomes criteria for CKD: eGFR categories G3–G5 and/or albuminuria categories A2–A3. ^h^In the English cohort, baseline eGFR was based on serum creatinine values recorded within the year before the index stroke using the Chronic Kidney Disease Epidemiology Collaboration formula. ^i^Defined as a prescription supply lasting until/past the date of the index stroke or ending within the previous 90 days

Supplementary Table 6. Incidence rates of recurrent ischemic stroke among the post-30-day cohorts. A, first year of follow-up. B, all follow-up

**A)**

|  | **England (n = 47,611)** | | | **Denmark (n = 58,223)** | | |
| --- | --- | --- | --- | --- | --- | --- |
|  | **Person-years** | **Events, n** | **IR per 100 person-years (95% CI)** | **Person-years** | **Events, n** | **IR per 100 person-years (95% CI)** |
| All | 39,221 | 2130 | 5.43 (5.20–5.67) | 49,650 | 3203 | 6.45 (6.23–6.68) |
| Sex |  |  |  |  |  |  |
| Females | 17,942 | 1063 | 5.92 (5.57–6.29) | 21,700 | 1453 | 6.70 (6.36–7.05) |
| Males | 21,279 | 1067 | 5.01 (4.72–5.32) | 27,951 | 1750 | 6.26 (5.97–6.56) |
| Age at index stroke, years |  |  |  |  |  |  |
| <65 | 12,884 | 514 | 3.99 (3.65–4.35) | 17,286 | 756 | 4.37 (4.07–4.70) |
| 65–75 | 11,332 | 515 | 4.54 (4.16–4.95) | 14,678 | 829 | 5.65 (5.28–6.05) |
| >75 | 15,005 | 1101 | 7.34 (6.91–7.78) | 17,686 | 1618 | 9.15 (8.71–9.61) |
| Time since index IS, months |  |  |  |  |  |  |
| <3 | 7722 | 766 | 9.92 (9.23–10.65) | 49,650 | 3203 | 6.45 (6.23–6.68) |
| 3–12 | 31,499 | 1364 | 4.33 (4.10–4.57) | 40,270 | 1999 | 4.96 4.75–5.19) |

**B)**

|  | **England (n = 47,611)** | | | **Denmark (n = 58,223)** | | |
| --- | --- | --- | --- | --- | --- | --- |
|  | **Person-years** | **Events, n** | **IR per 100 person-years (95% CI)** | **Person-years** | **Events, n** | **IR per 100 person-years (95% CI)** |
| All | 152,182 | 4774 | 3.14 (3.05–3.23) | 234,247 | 8138 | 3.47 (3.40–3.55) |
| Sex |  |  |  |  |  |  |
| Females | 68,049 | 2349 | 3.45 (3.31–3.59) | 102,183 | 3651 | 3.57 (3.46–3.69) |
| Males | 84,133 | 2425 | 2.88 (2.77–3.00) | 132,064 | 4487 | 3.40 (3.30–3.50) |
| Age at index stroke, years |  |  |  |  |  |  |
| <65 | 55,307 | 1080 | 1.95 (1.84–2.07) | 93,663 | 1992 | 2.13 (2.04–2.22) |
| 65–75 | 46,361 | 1272 | 2.74 (2.59–2.90) | 71,695 | 2247 | 3.13 (3.01–3.27) |
| >75 | 50,515 | 2422 | 4.79 (4.61–4.99) | 68,889 | 3899 | 5.66 (5.48–5.84) |
| Time since index IS, months |  |  |  |  |  |  |
| <3 | 39,221 | 2130 | 5.43 (5.20–5.67) | 49,650 | 3203 | 6.45 (6.23–6.68) |
| 3–12 | 112,961 | 2644 | 2.34 (2.25–2.43) | 40,270 | 1999 | 2.67 (2.60–2.75) |

*CI* confidence interval; *IR* incidence rate; *IS* ischemic stroke.

Supplementary Table 7. Cardiac disorders at baseline among patients included in the ischemic stroke cohort in the English (n = 52,419) and Danish (n = 62,501) cohorts

| **Characteristic, No. (%)** | **England (n = 52,419)** | **Denmark (n = 62,501)** |
| --- | --- | --- |
| Mechanical heart valve | NA | 20 (0.0) |
| Patent foramen ovale (undergone percutaneous transluminal closure) | 69 (0.1) | <5 |
| Cardiomyopathy | 333 (0.6) | 606 (1.0) |
| Endocarditis | 45 (0.1) | 145 (0.2) |
| Left ventricular thrombosis | 13 (0.0) | 7 (0.0) |
| Benign cardiac tumor (e.g., myxoma) | <5 | 9 (0.0) |
| Cardiac tumor | <5 | 11 (0.0) |
| Aorta surgery | NA | 48 (0.1) |

*NA* not applicable.
Numbers <5 and totals not presented to preserve anonymity.

Supplementary Fig. 1. Ascertainment of patients. A, English (n = 52,419) cohort. B, Danish (n = 62,501) cohort


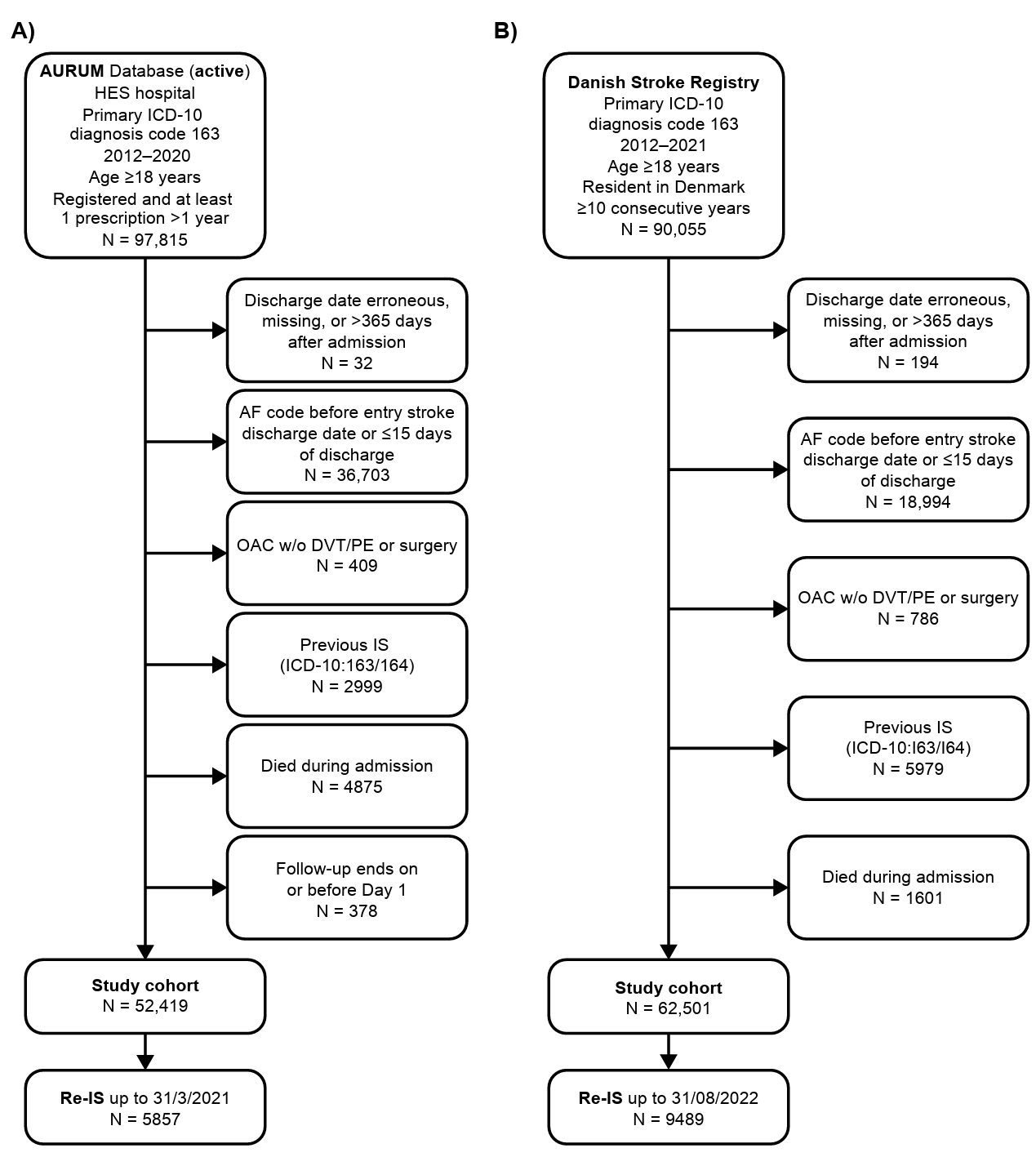


*AF* atrial fibrillation; *DVT* deep vein thrombosis; *HES* Hospital Episode Statistics; *ICD-10* International Classification of Diseases, Tenth Revision; *IS* ischemic stroke; *OAC* oral anticoagulant; *PE* pulmonary embolism; *Re-IS* recurrent ischemic stroke.

Supplementary Fig. 2. Crude cumulative hazard of recurrent IS by sex with 95% CI. A, English (n = 52,419) cohort. B, Danish (n = 62,501) cohort


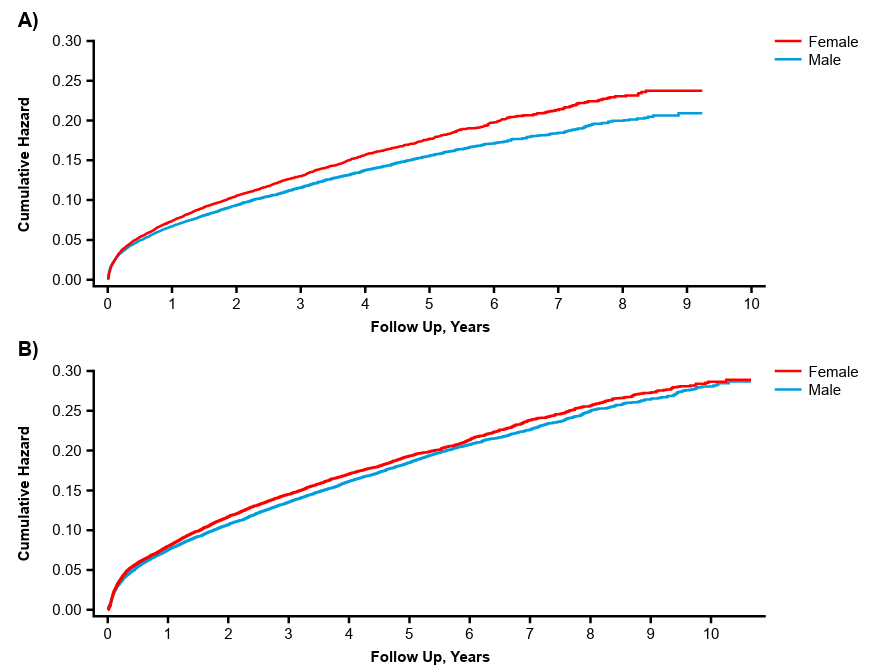
 *CI* confidence interval; *IS* ischemic stroke.

**Supplementary Fig. 3. Ascertainment of Patients from Day 30 Onward. A, English (n = 47,611) Cohort. B, Danish (n = 58,223) Cohort**

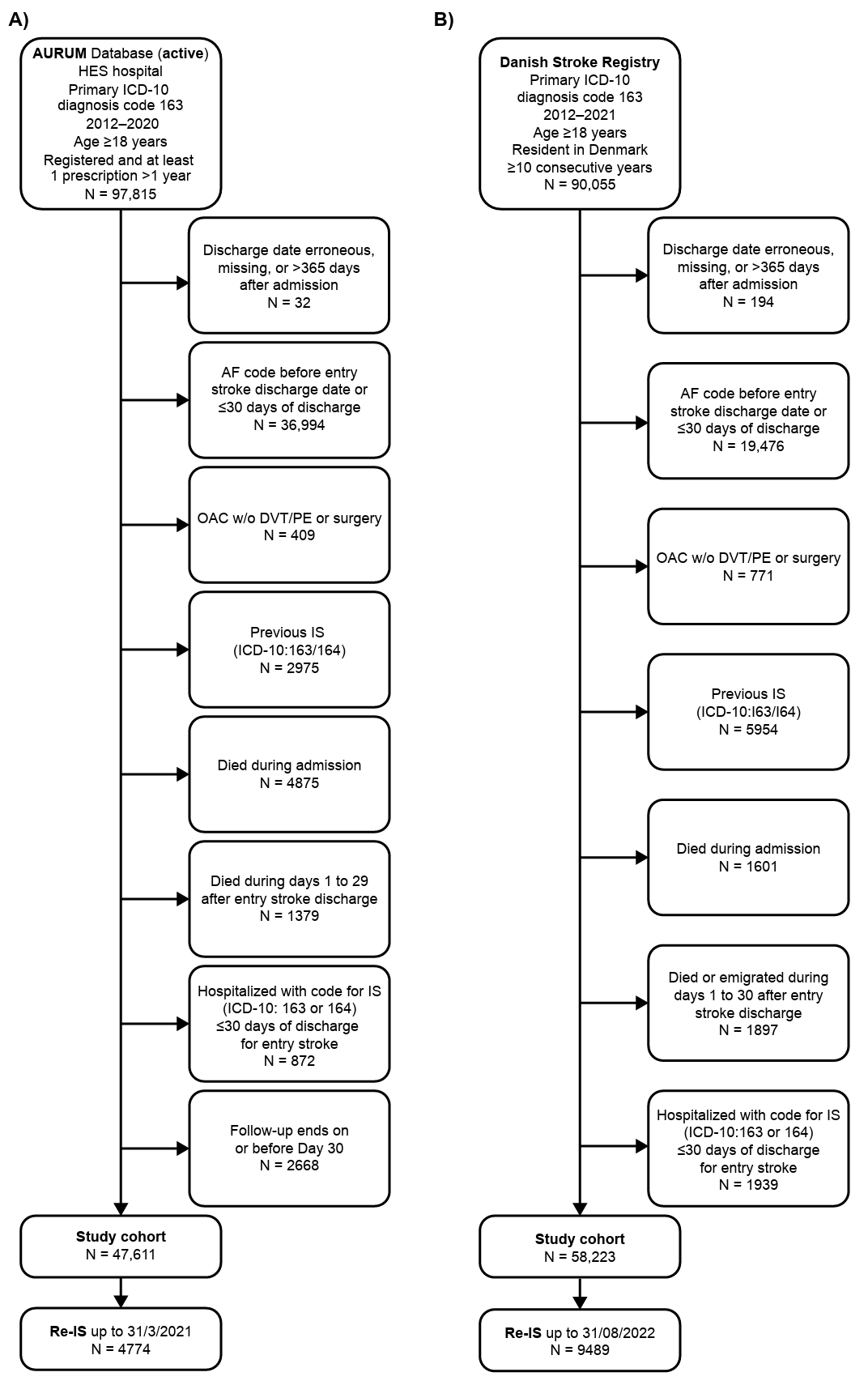


*AF* atrial fibrillation; *DVT* deep vein thrombosis; *HES* Hospital Episode Statistics; *ICD-10* International Classification of Diseases, Tenth Revision; *IS* ischemic stroke; *OAC* oral anticoagulant; *PE* pulmonary embolism; *Re-IS* recurrent ischemic stroke.

# References

1. Medicines and Healthcare Products Regulatory Agency. Clinical Practice Research Datalink Bibliography. <https://www.cprd.com/bibliography>. Accessed January 28, 2026

2. Padmanabhan S, Carty L, Cameron E, et al. (2019) Approach to record linkage of primary care data from Clinical Practice Research Datalink to other health-related patient data: overview and implications. Eur J Epidemiol 34(1):91-99. <https://doi.org/10.1007/s10654-018-0442-4>

3. Clinical Practice Research Datalink. CPRD Aurum May 2022 dataset (version 2022.05.001). <https://doi.org/10.48329/t89s-kf12>. Accessed September 30, 2025

4. Herbert A, Wijlaars L, Zylbersztejn A, et al. (2017) Data resource profile: Hospital Episode Statistics Admitted Patient Care (HES APC). Int J Epidemiol 46(4):1093-1093i. <https://doi.org/10.1093/ije/dyx015>

5. Gallagher AM, Dedman D, Padmanabhan S, et al. (2019) The accuracy of date of death recording in the Clinical Practice Research Datalink GOLD database in England compared with the Office for National Statistics death registrations. Pharmacoepidemiol Drug Saf 28(5):563-569. <https://doi.org/https://doi.org/10.1002/pds.4747>

6. Delmestri A, Prieto-Alhambra D (2020) CPRD GOLD and linked ONS mortality records: reconciling guidelines. Int J Med Inform 136:104038. <https://doi.org/10.1016/j.ijmedinf.2019.104038>

7. Schmidt M, Pedersen L, Sørensen HT (2014) The Danish Civil Registration System as a tool in epidemiology. Eur J Epidemiol 29(8):541-549. <https://doi.org/10.1007/s10654-014-9930-3>

8. Hindsholm MF, García Rodríguez LA, Brandes A, et al. (2024) Recurrent ischemic stroke in patients with atrial fibrillation while receiving oral anticoagulants. JAMA Neurol 81(8):805-813. <https://doi.org/10.1001/jamaneurol.2024.1892>

9. Wildenschild C, Mehnert F, Thomsen RW, et al. (2014) Registration of acute stroke: validity in the Danish Stroke Registry and the Danish National Registry of Patients. Clin Epidemiol 6:27-36. <https://doi.org/10.2147/clep.S50449>

10. Schmidt M, Schmidt SA, Sandegaard JL, et al. (2015) The Danish national patient registry: a review of content, data quality, and research potential. Clin Epidemiol 7:449-490. <https://doi.org/10.2147/clep.S91125>

11. Pottegård A, Schmidt SAJ, Wallach-Kildemoes H, et al. (2017) Data resource profile: the Danish national prescription registry. Int J Epidemiol 46(3):798-798f. <https://doi.org/10.1093/ije/dyw213>

12. Hald SM, Möller S, García Rodríguez LA, et al. (2021) Trends in incidence of intracerebral hemorrhage and association with antithrombotic drug use in Denmark, 2005-2018. JAMA Netw Open 4(5):e218380. <https://doi.org/10.1001/jamanetworkopen.2021.8380>

13. Helweg-Larsen K (2011) The Danish register of causes of death. Scand J Public Health 39(7 Suppl):26-29. <https://doi.org/10.1177/1403494811399958>

14. Johnsen SP, Ingeman A, Hundborg HH, et al. (2016) The Danish stroke registry. Clin Epidemiol 8:697-702. <https://doi.org/10.2147/clep.S103662>

15. Clegg A, Bates C, Young J, et al. (2016) Development and validation of an electronic frailty index using routine primary care electronic health record data. Age Ageing 45(3):353-360. <https://doi.org/10.1093/ageing/afw039>

16. Elhussein L, Robinson DE, Delmestri A, et al. (2024) Longitudinal trajectories of frailty are associated with short-term mortality in older people: a joint latent class models analysis using 2 UK primary care databases. J Clin Epidemiol 173:111442. <https://doi.org/10.1016/j.jclinepi.2024.111442>

17. Govan L, Langhorne P, Weir CJ (2009) Categorizing stroke prognosis using different stroke scales. Stroke 40(10):3396-3399. <https://doi.org/10.1161/strokeaha.109.557645>
